# *Caenorhabditis Elegans*

**DOI:** 10.1002/(SICI)1097-0061(200004)17:1<37::AID-YEA11>3.0.CO;2-P

**Published:** 2000


**Yeast**
*Yeast* 2000; **17**: 37–42.

Featured Organism

### *Caenorhabditis elegans*

### Summary

- **Transparent, free-living nematode worm.**
- **Unsegmented body plan with full set of differentiated tissues (neural, endoderm, ectoderm and muscle).**
- **Genome size ~97 Mb, as five autosomes and one X sex chromosome.**
- **Fully sequenced genome, which comprises ~20 000 predicted genes.**
- **Defined cell lineage.**
- **Has made major contribution to studies of development, cell-to-cell signalling, cell ageing and cell death processes.**
- **Large-scale gene deletion, microarray analysis of gene expression and two-hybrid protein interaction analysis projects under way.**
- **Comparative studies mainly with *C. briggsae*, but also with other free-living and parasitic nematodes.**

### Background

*C. elegans* (Figure 1) is a free-living, terrestrial worm, which grows to about 1 mm in length. It is a member of the phylum Nematoda. Nematodes are smooth-skinned, unsegmented worms with a long cylindrical body shape which tapers at the ends. This phylum is made up of the roundworms and threadworms, which may be free-living or parasitic, aquatic or terrestrial. Nematodes are very abundant in nature, and include important human, animal and plant parasites.

*C. elegans* lives in the soil, especially amongst rotting vegetation, feeding on microbes such as bacteria. Although it is a primitive organism, it does share many biological characteristics with humans. Two sexes of *C. elegans* exist, a self-fertilizing hermaphrodite and a more rare male. The female is a self-fertilizing protandrous hermaphrodite, producing first sperm and then oocytes. Mating between males and females produces mainly progeny as the male cross-sperm outcompete the hermaphrodite's. The development of the nematode from the single cell that is formed is a complex process. Within the egg, a period of cell multiplication is followed by morphogenesis and differentiation, and the nematode hatches as a first stage larva. There are four larval stages, punctuated by moults, before the reproductive adult. After reproduction, the parent begins to age; this is marked by a loss of vigour and is followed by death, the average life span being only 2–3 weeks.

The body of the nematode is transparent, permitting observation of all of its cells. The first stage larva has 558 cells, while the adult female has 959 (1031 in the male). The lineage of each of these somatic cells is essentially invariant between individual worms. In the hermaphrodite hatchling first stage larva, 302 of these somatic cells are neurons, and 81 are muscle cells. The *C. elegans* nervous system consists of a ring of nerves forming a primitive brain, a ventral nerve cord running down

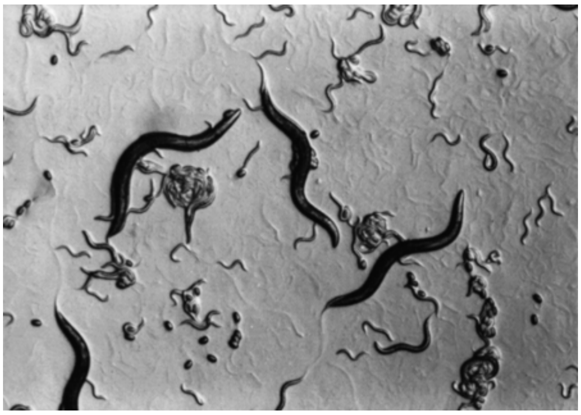

**Figure 1.** *Caenorhabditis elegans*. Courtesy of Henri G. A. M. van Luenen, The Netherlands Cancer Institute, Amsterdam, The Netherlands

Copyright © 2000 John Wiley & Sons, Ltd.

the body and a smaller dorsal nerve cord. There are sense organs in the head and tail that respond to tastes and smells and sense temperature variation and touch. *C. elegans* has no eyes, but it may have some small response to light. It moves using four longitudinal bands of muscle paired subdorsally and subventrally, alternate flexing and relaxation of these muscles causes dorsal-ventral waves along the body.

### Sources

Dr Mark Blaxter's web page: 'Genomics course notes' (see Web-based resources). Professor Donald Riddle's Group web page: 'An introduction to *C. elegans*' (see Web-based resources).

### Tools for study

Several markers exist for use in tracking the uptake of a plasmid or integration of an extrachromosomal array, the most popular being the *rol-6* dominant allele in plasmid pRF4. Plasmids are microinjected into the syncytial gonad arms of hermaphrodite nematodes and the F1 progeny can be scored about 3 days after injection, unless the phenotype of the marker is stage-specific in its expression.

In addition to the functional analysis techniques detailed below, genetic screens for enhancers and suppressors of particular phenotypes are still in common use. There are over 2500 loci known by mutation, and the *Caenorhabditis* Genetics Centre (see Web-based resources) has a large collection of strains carrying these mutations.

A library of strains containing single cosmid extrachromosomal arrays has been made available (Janke *et al.*, 1997) for functional mapping by rescue of mutant phenotypes.

A selection of gene inactivation and deletion strategies are available, ranging from deletion by targeted insertion of Tc1 transposons followed by sib selection, to transient loss of function by RNA interference, to random, UV-mediated chemical mutagenesis, followed by PCR screening. Detailed explanations of all these techniques can be found on the Comprehensive Protocols Collection web site (see Web-based resources).

The Sanger Center is collaborating in a gene knockout consortium with the Oklahoma Medical Research Foundation, the University of British Columbia, Dr Ralf Baumeister's lab at the Ludwig-Maximilians-Universität, Dr Ronald Plasterk's lab at The Netherlands Cancer Institute, and Dr Yuji Kohara's lab at the National Institute of Genetics (see the interview with Alan Coulson and Patricia Kuwabara, in this issue). The participants are currently optimizing the latter gene deletion method, prior to accepting requests from the community for genes to be targeted.

Being transparent, *C. elegans* is ideal for use with $\beta$-galactosidase gene fusions and *in situ* detection systems for the analysis of expression patterns of genes. Web pages containing the results of two projects using these techniques are listed in the Web-based resources section below. A series of vectors for expression of GFP gene fusions (Miller *et al.*, 1999) are also being used to study gene expression in the worm.

The Stanford University Medical Centre is hosting the Worm Microarray Centre. Dr Stuart Kim has called for worm labs to supply RNA samples for hybridization to the chips. The original chip contained DNA fragments representing 11 990 genes; 155 experiments were done using this chip and the data from over 100 of these experiments has been used in clustering analysis. A new whole-genome chip has been produced, which contains ~1 kb long, exon-rich fragments representing 20 000 genes. The project is overseen by a panel of researchers from six different research institutions, including Patricia Kuwabara (see Interview, in this issue).

Following a successful pilot experiment using genes involved in vulval development, a genome-wide two-hybrid protein interaction mapping project is under way (Walhout *et al.*, 2000).

The Washington University Genome Sequencing Centre has started sequencing the *C. briggsae* genome and has produced ~8.7 Mb of sequence so far. This sequence will be a powerful tool for comparative genomics with *C. elegans*.

### Web-based resources

Presented here are a selection of the best web pages providing *C. elegans* resources.

### Databases

The Sanger Centre *C. elegans* pages
**http://www.sanger.ac.uk/Projects/C_elegans/**
This page has details of the Sanger Centre contribution towards the *C. elegans* sequencing project,

Copyright © 2000 John Wiley & Sons, Ltd. 

including the Science paper in which the complete sequence was published:
**http://www.sanger.ac.uk/Projects/C_elegans/Science98/**

BLAST searches of the sequence data, several versions of ACeDB (A *C. elegans* DataBase) and the database of predicted proteins, Wormpep18, are made available on the site. Gene expression pattern data is also available as part of ACeDB, although most web versions do not allow views of the micrographs.

The pages also contain details of the *C. elegans* gene knockout consortium. Further information on this project, including a searchable list of the genes already targeted, can be found at the site of the Centre for Integrated Genomics:
**http://www.cigenomics.bc.ca/elegans/**

The Washington University Genome Sequencing Centre
**http://genome.wustl.edu/gsc/index.shtml**
**http://genome.wustl.edu/gsc/C_elegans/Science98/**
The centre was responsible for providing half of the *C. elegans* genome sequence and provides access to ACeDB at this site. This page also contains details of all the other contributions of the centre to genome and EST sequencing projects. They have also started sequencing of *C. briggsae* and have currently produced ~8.7 Mb of sequence.
**http://genome.wustl.edu/gsc/Projects/briggsae.shtml**

The *C. elegans* Database
**http://stein.cshl.org/elegans/**
This is an alternative version of ACeDB which has been modified to allow views of the expression pattern micrographs. Curator: Lincoln Stein (Cold Spring Harbor Laboratory, New York, USA).

WormPD and YPD
**http://www.proteome.com/databases/index.html**
A commercial page produced by Proteome Inc. which provides a database containing all of the currently known information on each worm protein, from the effects of deletion to literature listings (Costanzo *et al.*, 2000). The results of BLAST searches against *C. elegans* proteins, human proteins and proteins from model organisms, such as *S. cerevisiae*, are listed for each protein. The database is freely available to academic users; corporate users should direct enquiries regarding subscription services to **hfo@proteome.com.**

The Worm DNA Microarray Center
**http://cmgm.stanford.edu/~kimlab/wmdirectorybig.html**
These pages contain details of a large-scale, chip-based, gene expression analysis project for *C. elegans*, run by the laboratory of Stuart Kim, in collaboration with several other laboratories. The pages include information on how to take part and how to analyse the data once the chosen experiments have been performed. Clustering analysis has been done on the data obtained from ~100 experiments and a table of the results is available.

The expression pattern database
**http://www.personal.leeds.ac.uk/~acedb/Hope/epa.htm**
Dr Ian Hope's Lab web page contains a regularly updated listing of the expression patterns of individual *C. elegans* genes determined by use of lacZ fusion constructs (Hope *et al.*, 1996). Authors: Petra Bauer, Andrew Mounsey and Ian Hope (Leeds University, UK).

NEXTDB—The Nematode Expression Pattern Database
**http://watson.genes.nig.ac.jp/db/index.html**
Dr Yuji Kohara's web page is a searchable database of expression patterns of *C. elegans* genes as determined by *in situ* hybridization on whole mount embryos, with pictures provided for different stages of development (Tabara *et al.*, 1996).

Caenorhabditis Genetics Centre
**gopher://elegans.cbs.umn.edu/**
The centre acquires, maintains and distributes a collection of over 3000 *C. elegans* genetic stocks. The centre is also responsible for the production of the Worm Breeders' Gazette, which can be viewed on the web by following the link from The *C. elegans* WWW page, which is described below. Curator: Theresa L. Stiernagle.

### General information

The *C. elegans* WWW page
**http://elegans.swmed.edu/**
This site has a UK mirror:
**http://www.c.elegans.leeds.ac.uk/index.shtml**
This incredibly comprehensive page includes listings of worm labs, literature, meetings and archives of

Copyright © 2000 John Wiley & Sons, Ltd.

postings to the bionet.celegans newsgroup. Author: Leon Avery, Mirror curator: David Coates.

The Comprehensive Protocol Collection—Worm protocols
**http://www.dartmouth.edu/artsci/bio/ambros/protocols/worm_protocols.html**
A catalogued collection of worm protocols, written and contributed by experts, with in-depth practical comments and tips. An invaluable resource for anyone setting up a worm lab or trying out a new technique.

The Mark Blaxter lab web page
**http://www.ed.ac.uk/~mbx/C_elegans_genome/Celegansgenome.html**
**http://www.ed.ac.uk/~mbx/C_elegans/Ce_intro.html**
An introduction to *C. elegans* biology, and an overview of the pattern and process behind the genome sequence. These pages form an excellent resource of genome information for those not working on nematodes. Author: Mark Blaxter.

Professor Donald Riddle's Group web page
**http://www.biotech.missouri.edu/Dauer-World/Wormintro.html**
In addition to an extensive collection of information about the Dauer form of *C. elegans*, this page has an introduction to *C. elegans* for those not familiar with it.

### Current status of genome/knowledge

*C. elegans* is diploid and has five pairs of autosomal chromosomes (named I, II, III, IV and V) and a pair of sex chromosomes (X). XX worms are hermaphrodite, XO worms are male.

The essentially complete genome sequence of *C. elegans* was released in 1998 (The *C. elegans* Sequencing Consortium, 1998). It was the second eukaryote genome and the first animal genome to be completely sequenced. ACeDB, the database engine now used by many genome sequencing consortia as the database of choice, was originally developed for the *C. elegans* genome project.

The current release of the genome sequence (25/11/1999) has 96 893 008 bp. The sequence error, which was estimated from overlaps between cosmids and resequencing of clones in the two sequencing centres, was found to be <1 in 10 000 bases (probably nearer to 1 in 100 000). The genome encodes over 19 000 genes, the average length of a gene being 5 kb with an average of 5 exons per gene. 1341 proteins have been experimentally characterized, 10 041 genes encode proteins with homology to characterized proteins and there still remain 8012 proteins whose function is designated 'unknown' in WormPD (see Web-based resources).

### Future aims

Ian Hope (University of Leeds, UK) generates expression pattern data using lacZ and GFP gene fusions (see Web-based resources). He thinks that this type of data (whether it be produced by reporter gene fusion or *in situ* hybridization methods—see Web-based resources, Yuji Kohara's lab page) will become very important in the future, for understanding how the genome generates the animal. He feels that the information produced by these techniques will complement data generated by forward and reverse genetics approaches and that it should be integrated with other data on *C. elegans*, as has been started in ACeDB. In his view, microarray data is a powerful method for generating temporal expression data, but for the time being is more limited for provision of the spatial information that *in situ* methods can generate. He sees the handling, presentation and utilization of expression data as easier for microarray data than the *in situ* data and this may be a problem area to be addressed in the future. Expression data is composed of time and space and level of expression, which are continuua and cannot easily be digitized. Even though *C. elegans* has an invariant cell lineage, which would somewhat simplify the digitization of the data with respect to space and time, this would still be a lot of work. Expression pattern data handling will present problems for most, if not all, databases and he feels that a 'virtual' option may be the answer, in which a 'virtual worm' is built around the expression pattern data and genetic regulatory systems by which each gene is produced at the appropriate time, place and level. This could, perhaps, even be used to perform virtual experiments in the far future. As yet, gene fusion and *in situ* techniques cannot be performed in the same high-throughput manner as the microarray experiments are, so, unfortunately, despite the ongoing

Copyright © 2000 John Wiley & Sons, Ltd.

advances in virtual technology, we may have to wait some time for a virtual worm.

Robert Barstead (OMRF, Oklahoma City, USA) is head of one of the groups participating in the *C. elegans* knockout project. He points out that the benefit to be gained from the project will depend largely on the efforts of the users, once they receive the mutants, in terms of making sure that each mutant contains a clean deletion and making a thorough hunt for a phenotype. He feels that it will be necessary to develop more sophisticated ways to assess phenotype. Minor effects might only be observed using computer-aided measurement and phenotypes with low penetrance may only be detected upon analysis of a population, perhaps using computerized, artificial vision systems. He also suggests that a systematic, collaborative effort to apply the same set of phenotypic assays to all strains would aid in the collation and comparison of data, allowing the discovery of correlations that might be invisible to individual groups. This would also facilitate the comparison of data produced by large-scale projects with those generated on an *ad hoc* basis, which can often be a stumbling block. He, too, is aware of the need for development of new informatics tools to store, represent and mine all the information being generated about *C. elegans*, and points out that the developers of ACeDB are currently working to improve the system. In his opinion, microarray data is at its most valuable when comparisons with other experiments or across sets of genes can be made, hence he is very much in favour of the centralized service provided at Stanford.

Stuart Kim (Stanford University, USA) is in charge of the centralized *C. elegans* microarray centre at Stanford. The data produced, which is stored in a database at Stanford, is kept private for a limited time, after which it is made public. These experiments frequently light up too many genes for one group to investigate, so there are plenty of target genes and downstream applications of the data to keep everyone busy. In his view, it is by doing several carefully thought-out experiments and using the powerful data analysis approaches that the best candidates are identified. An example of this is clustering analysis, which groups genes whose expression is regulated in the same way across several experiments. This can be used to assign a gene of previously unknown function to a pathway, or to enable the discovery of new transcription factor binding sites. They are also using the array with RNA probes obtained from mutants which lack certain tissues and also with samples from mutants which have too much of a tissue, which gives data on gene expression in the tissue of interest. This approach has already borne fruit, identifying 1400 genes that are expressed in the germline. They plan to follow up this result with a study of 766 of the genes that they have shown to be expressed in the germline. They and their collaborators plan to use RNA interference for loss-of-function studies, to do further microarray expression analyses and two-hybrid analyses to determine protein interaction partners. Stuart also collaborates with David Botstein and Pat Brown, using the same system and database. They are employing programmers and statisticians in a bid to generate a better way to store and analyse the enormous amounts of data they generate. Stanford alone plans to produce a terabyte of data this year; this requires a more complex, Oracle-based database, rather than one based on a flat file system.

### References

Costanzo MC, Hogan JD, Cusick ME, *et al.* 2000. The Yeast Proteome Database (YPD) and *Caenorhabditis elegans* Proteome Database (WormPD): comprehensive resources for the organization and comparison of model organism protein information. *Nucleic Acids Res* **28**(1): 73–76.

Hope IA, Albertson DG, Martinelli SD, Lynch AS, Sonnhammer E, Durbin R. 1996. The *C. elegans* expression pattern database—a beginning. *Trends Genet* **12**: 370–371.

Janke DL, Schein JE, Ha T, *et al.* 1997. Interpreting a sequenced genome: toward a cosmid transgenic library of *Caenorhabditis elegans*. *Genome Res* **7**: 974–985.

Miller DM, Desai NS, Hardin DC, *et al.* 1999. Two-color GFP expression system for *C. elegans*. *Biotechniques* **26**: 914.

Tabara H, Motohashi T, Kohara Y. 1996. A multi-well version of *in situ* hybridization on whole mount embryos of *Caenorhabditis elegans*. *Nucleic Acids Res* **24**(11): 2119–2124.

The *C. elegans* Sequencing Consortium. 1998. Genome sequence of the nematode *Caenorhabditis elegans*. A platform for investigating biology. *Science* **282**: 2012–2018.

Walhout AJM, Sordella R, Lu X, *et al.* 2000. Protein interaction mapping in *C. elegans* using proteins involved in vulval development. *Science* **287**: 116–122.

Copyright © 2000 John Wiley & Sons, Ltd. 

*Comparative and Functional Genomics* is a cross-organism journal, publishing studies on complex and model organisms. The Featured Organism article aims to present an overview of an organism, primarily for those working on other systems. It provides background information on the organism itself and on genomics studies currently in progress. It also gives a list of websites containing further information and a summary of the status of the study of the genome. These sections are a personal critical analysis of the current studies of the particular organism. The 'Future aims' section is intended to be of interest to readers who work on the chosen organism and those who study other systems, and the opinions expressed therein are those of the named contributors.
This article was written by Dr Joanne Wixon (Managing Editor).

Many thanks to Dr Mark Blaxter for his assistance with the summary and background sections and to Dr Ian Hope, Dr Robert Barstead and Dr Stuart Kim for sharing their thoughts on the future of *C. elegans* genomics research.

**Dr Mark Blaxter**
Institute of Cell, Animal and Population Biology, Ashworth laboratories, 311, King's Buildings, University of Edinburgh, West Mains Road, Edinburgh EH9 3JT, UK

**Dr Ian Hope**
School of Biology, The University of Leeds, Leeds LS2 9JT, UK

**Dr Robert Barstead**
Assistant Member, Oklahoma Medical Research Foundation, Department of Molecular and Cell Biology, 825 NE 13th Street, Oklahoma City, OK 73104, USA. E-mail: barsteadr@omrf.ouhsc.edu

**Dr Stuart Kim**
Department of Developmental Biology, 279 Campus Drive, Stanford University Medical Center, Stanford, CA 94305–5329, USA

Copyright © 2000 John Wiley & Sons, Ltd. 
